# Elevated high-density lipoprotein in adolescents with Type 1 diabetes is associated with endothelial dysfunction in the presence of systemic inflammation

**DOI:** 10.1093/eurheartj/ehz114

**Published:** 2019-03-12

**Authors:** Scott T Chiesa, Marietta Charakida, Eve McLoughlin, Helen C Nguyen, Georgios Georgiopoulos, Laura Motran, Yesmino Elia, M Loredana Marcovecchio, David B Dunger, R Neil Dalton, Denis Daneman, Etienne Sochett, Farid H Mahmud, John E Deanfield

**Affiliations:** 1 Vascular Physiology Unit, UCL Institute of Cardiovascular Science, London, UK; 2 1st Cardiology Clinic, University of Athens, Hippokratio Hospital, Athens, Greece; 3 Department of Pediatrics, Hospital for Sick Children, University of Toronto, Toronto, Canada; 4 Department of Paediatrics, University of Cambridge, Cambridge, UK; 5 Institute of Metabolic Science, University of Cambridge, Cambridge, UK; 6 WellChild Laboratory, St. Thomas’ Hospital, King’s College London, London, UK

**Keywords:** Type 1 diabetes, Adolescents, HDL function, Endothelial function, Inflammation

## Abstract

**Aims:**

High-density lipoprotein (HDL) function may be altered in patients with chronic disease, transforming the particle from a beneficial vasoprotective molecule to a noxious pro-inflammatory equivalent. Adolescents with Type 1 diabetes often have elevated HDL, but its vasoprotective properties and relationship to endothelial function have not been assessed.

**Methods and results:**

Seventy adolescents with Type 1 diabetes (age 10–17 years) and 30 age-matched healthy controls supplied urine samples for the measurement of early renal dysfunction (albumin:creatinine ratio; ACR), blood samples for the assessment of cardiovascular risk factors (lipid profiles, HDL functionality, glycaemic control, and inflammatory risk score), and had their conduit artery endothelial function tested using flow-mediated dilation (FMD). HDL-c levels (1.69 ± 0.41 vs. 1.44 ± 0.29mmol/L; *P* < 0.001), and glycated haemoglobin (HbA1c) (8.4 ± 1.2 vs. 5.4 ± 0.2%; *P* < 0.001) were increased in all patients compared with controls. However, increased inflammation and HDL dysfunction were evident only in patients who also had evidence of early renal dysfunction (mean ± standard deviation for high-ACR vs. low-ACR and healthy controls: inflammatory risk score 11.3 ± 2.5 vs. 9.5 ± 2.4 and 9.2 ± 2.4, *P* < 0.01; HDL-mediated nitric-oxide bioavailability 38.0 ± 8.9 vs. 33.3 ± 7.3 and 25.0 ± 7.7%, *P* < 0.001; HDL-mediated superoxide production 3.71 ± 3.57 vs. 2.11 ± 3.49 and 1.91 ± 2.47nmol O_2_ per 250 000 cells, *P* < 0.05). Endothelial function (FMD) was impaired only in those who had both a high inflammatory risk score and high levels of HDL-c (*P* < 0.05).

**Conclusion:**

Increased levels of HDL-c commonly observed in individuals with Type 1 diabetes may be detrimental to endothelial function when accompanied by renal dysfunction and chronic inflammation.

**Figure:**
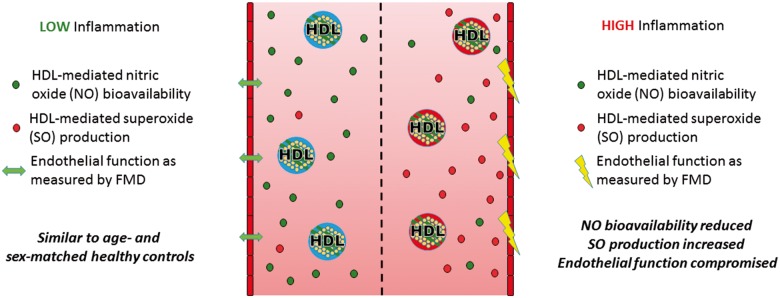

## Introduction

Cardiovascular disease (CVD) accounts for more than 60% of all deaths in patients with Type 1 diabetes from middle-age onwards, with females at particular risk.^1^ The development of Type 1 diabetes in the first decade of life carries a three-fold greater risk of CVD than its diagnosis in the 3rd decade,^2^ suggesting that adolescence is a critical period in the development of these complications. In support of this, previous research has shown the transition through puberty to be accompanied by a deterioration in glycaemic control, increased exposure to CVD risk factors, and the appearance of the first signs of subclinical damage.^3^^,^^4^ Identification of the underlying mechanistic pathways responsible for these early vascular changes is therefore vital in order to inform potential intervention strategies.

One of the earliest detectable indicators of vascular damage is endothelial dysfunction. This subclinical marker—which can be measured non-invasively using flow-mediated dilation (FMD)—is an established predictor of future adverse CVD outcomes.^5^ In agreement with previous studies,^6^^,^^7^ recent work from our group, as part of the Adolescent Type 1 Diabetes cardio-renal Intervention Trial (AdDIT), has reported the presence of endothelial dysfunction in Type 1 diabetes as early as the second decade of life (average age 14 years; duration of disease 7 years).^4^ In addition, we have also noted an increase in the serum concentration of high-density lipoprotein cholesterol (HDL-c), a phenomenon common in Type 1 diabetes.^8–11^

High-density lipoprotein (HDL) is widely-considered to be a vasoprotective particle due to its many anti-atherogenic and anti-inflammatory properties and is associated with improved CVD outcomes in epidemiological studies.^12^ HDL levels in Type 1 diabetes are commonly reported to be approximately 0.25 mmol/L higher than those observed in the general population,^13^ yet CVD risk in this patient group is considerable. We and others have demonstrated the capacity for HDL to transform from a protective anti-inflammatory molecule to a dysfunctional pro-inflammatory equivalent in the presence chronic disease.^14–17^ This effect is mediated through factors such as systemic inflammation^17^ or renal dysfunction,^18^ and can result in a reversal of the well-documented inverse relationship between circulating HDL levels and adverse CVD outcomes.^19^

We have also shown pro-inflammatory biomarkers and cytokines to be elevated in patients with Type 1 diabetes even at this young age, suggestive of a ‘chronic inflammatory’ response,^20^^,^^21^ as well as our ability to identify patients at high-risk of cardio-renal complications through the detection of early changes in renal function measured using of a standardized albumin-creatinine algorithm adjusted for age, sex, and duration of disease.^22^ In a subset of adolescents with Type 1 diabetes approached to take part in AdDIT, we therefore sought to investigate (i) whether the endothelial-protective properties of HDL (HDL-mediated endothelial NO bioavailability, SO production, and PON-1 activity) are impaired in this patient group, (ii) whether potentially modifiable risk factors (e.g. glycaemic control, renal dysfunction, chronic inflammation) are associated with these changes, and (iii) whether the presence of increased levels of dysfunctional HDL-c are associated with reduced endothelial function.

## Methods

### Study participants

Seventy patients with Type 1 diabetes were recruited from those screened for participation in the multi-centre international Adolescent Type 1 Diabetes cardio-renal Intervention Trial (AdDIT) in Toronto, Canada; alongside 30 healthy controls. Full details of inclusion and exclusion criteria for AdDIT have been previously published,^23^ and detailed information on participant selection and power calculations for the current study can be found in the Supplementary material online, *file*. Informed written consent was obtained from each participant aged 16 years and above, and those <16 years old provided personal assent to take part alongside the provision of written informed consent by their legal guardian/caregiver. All procedures were approved by The Hospital for Sick Children Research Ethics Board, Toronto, Canada and conformed to the Declaration of Helsinki.

### Urine analyses

Two sets of three consecutive early morning first-void urine samples were collected in patients with Type 1 diabetes for the measurement of albumin:creatinine ratio (ACR), as described previously.^22^

### Blood analyses

Serum samples for measurement of lipid profiles, inflammatory cytokines/chemokines, cystatin C, and HDL functionality were drawn in the fasted state, immediately centrifuged at 3000 rpm for 10 min, and separated into 0.5 mL aliquots for freezing at −80°C. Lipid profiles (total cholesterol, LDL-c, HDL-c, non-HDL-c, triglycerides) and glycated haemoglobin (HbA1c) was measured using routine laboratory techniques. Inflammatory markers were measured using The Discovery Human Cytokine Array/Chemokine Array 41-Plex Assay^®^ (Eve Technologies Corp, Calgary, Canada), used to simultaneously quantify 41 cytokine/chemokine/biomarkers using the Bio-Plex™ 200 system (Bio-Rad Laboratories, Inc., Hercules, USA) and a Milliplex human cytokine kit (Millipore, St. Charles, USA).^20^ Details of all cytokines/chemokines included in these assays can be found in Supplementary material online, *Table S1*.

### HDL function analyses

#### HDL isolation

HDL was isolated by sequential ultracentrifugation (*d* = 1.063 − 1.21 g/mL) using solid potassium bromide for density adjustment.^24^ After isolation, the amount of HDL recovered was quantified and expressed in µg of protein. HDL was stored at 4°C and functional assays were carried out in duplicate within 2 weeks of isolation.

#### HDL functionality

Functional properties of HDL were assessed using measures of HDL-mediated endothelial nitric oxide (NO) bioavailability, superoxide (SO) production, and serum paraoxonase (PON-1) activity. Full methodology for these measures can be found in the Supplementary material online, *file*, and details on the reproducibility and biological variability of each of these assays have been previously described.^25^

### Endothelial function analysis

Endothelial function was assessed using FMD in a temperature-controlled room. Full details of the technique are available in the Supplementary material online, and reproducibility and biological variability have been previously published.^26^

### Calculations

In order to characterize early changes in renal function, patients with Type 1 diabetes were split into high-risk (upper tertile) and low-risk (middle and lower tertile) groups based on a standardized log-transformed ACR adjusted for age, sex, and duration of disease. This algorithm, derived from previous longitudinal data,^22^ has previously been shown in multiple cohorts to stratify risk for multiple early cardio-renal complications.^4^^,^^22^^,^^23^^,^^27^ In order to quantify inflammatory burden, an inflammatory risk score was created using a combination of five pro-inflammatory markers [epidermal growth factor (EGF), chemokine growth-regulated oncogene (GRO), platelet-derived growth factor AA (PDGF-AA), platelet-derived growth factor BB (PDGF-BB), and soluble CD-40 ligand (sCD40L)] chosen *a priori* based on previous research from our group showing their elevation in adolescents with Type 1 diabetes.^20^ To create the score, each inflammatory marker was categorized into tertiles (low = 1, middle = 2, high = 3), with these values then summed to create an overall value (min. 5, max. 15). The combined impact of HDL and inflammation levels on endothelial function were assessed by converting HDL and inflammatory scores to dichotomous variables based on their median values, and then combining these into one of four groups: low HDL/low inflammation, low HDL/high inflammation, high HDL/low inflammation, and high HDL/high inflammation. Body mass index (BMI) *z*-scores were calculated using the lambda-mu-sigma (LMS) method. HbA_1c_ (mmol/mol) was calculated using the formula [(HbA_1c_ (%) × 10.93) − 23.5]. Estimated glomerular filtration rate (eGFR) was calculated using the Zappitelli combined formula [507.76 × e^0.003 × height^)/(Cystatin C0.635 × Creatinine ^0.547^)], which has previously been shown to accurately eGFR in paediatric populations.^28^

### Statistical analyses

The primary endpoint for this study was to investigate differences in HDL functionality between ACR groups, with secondary outcomes aimed at identifying risk factors associated with these changes, and investigating whether an interaction of these factors with HDL levels was associated with changes in endothelial function. Descriptive data are expressed as mean ± standard deviation or median (interquartile range) for normally and non-normally distributed data, respectively. Normal distribution was assessed using the Shapiro–Wilk test and graphical inspection of histograms and normality plots. Comparisons between healthy controls and patients with Type 1 diabetes were conducted using independent samples *t*-test or Kruskall–Wallis test. General or generalized linear models were implemented to calculate mean differences and 95% confidence intervals in variables of interest between ACR subgroups. Pearson χ^2^ test was used to test for different distributions of sex and ACR risk-group within combined HDL/inflammation groups. One way ANOVA with *post* *hoc* LSD testing was used to assess differences in HDL function between combined HDL/inflammation groups. Multiple linear regression was used to identify independent predictors of HDL function, as well as relationships between combined HDL/inflammation groups and FMD following adjustments for potential confounding factors (age, sex, BMI *z*-score, eGFR, and systolic blood pressure). All cytokine/chemokine measurements were log transformed prior to analysis in statistical models. All statistical analyses were carried out using SPSS v.22.0, and two-sided *P* levels of 0.05 were considered statistically significant.

## Results

### Participant characteristics

Demographic and clinical characteristics for all participants are shown in *Table 1*. There were no differences in age, sex distribution, blood pressure, BMI *z*-scores, or the majority of measured lipids (LDL-c, non-HDL-c, triglycerides) between healthy controls and adolescents with Type 1 diabetes (*P* > 0.05 for all). In contrast, HDL-c (1.67 ± 0.38 vs. 1.44 ± 0.29mmol/L; *P* < 0.001); HbA_1c_ (8.3 vs. 5.4%; *P* < 0.001); and inflammatory risk score (10.4 ± 2.6 vs. 9.2 ± 2.4; *P* = 0.030) were elevated in Type 1 diabetes compared with controls. When stratifying by ACR groups, increases in inflammatory risk score were strongest in adolescents with Type 1 diabetes who also displayed evidence of early renal dysfunction (high-ACR group), with all five pro-inflammatory markers found to be elevated in this group alone (*P* < 0.05 for all; data not shown). As a result, only adolescents with Type 1 diabetes and high-ACR had a higher inflammatory score, compared with both low-ACR patients and healthy controls (11.3 ± 2.4 vs. 9.5 ± 2.4 and 9.2 ± 2.5 for high-ACR group vs. low-ACR group and controls, respectively; *P* < 0.05 for high vs. other groups; *Table 2*).

**Table 1:** Participant characteristics for control and Type 1 diabetes groups

	Healthy controls	Type 1 diabetes	*P*-value
*n*	30	70	
Demographics			
Age (years)	13.9 ± 2.1	14.6 ± 1.7	0.103
Sex (% male)	53	39	0.184
BMI *z*-score	0.47 ± 0.95	0.70 ± 0.80	0.262
Type 1 diabetes associated risk factors			
Disease duration (years)	—	8.9 ± 3.8	**—**
HbA_1c_ (% )	5.4 (5.3–5.5)	8.3 (7.8–9.1)	**<0.001**
eGFR (mL/min/1.73 m^2^)	116 ± 18	125 ± 21	**0.048**
Blood pressure			
SBP (mmHg)	111 ± 11	115 ± 10	0.073
DBP (mmHg)	66 ± 8	67 ± 7	0.670
MAP (mmHg)	81 ± 8	83 ± 6	0.258
Lipids			
HDL-c (mmol/L)	1.44 ± 0.29	1.67 ± 0.38	**0.001**
Non-HDL-c (mmol/L)	2.65 ± 0.55	2.59 ± 0.63	0.617
LDL-c (mmol/L)	2.23 ± 0.51	2.20 ± 0.58	0.743
Triglycerides (mmol/L)	0.75 (0.63–1.31)	0.78 (0.62–0.95)	0.422
HDL function			
NO bioavailability (% change from buffer-treated cells)	38.0 ± 8.9	29.5 ± 8.5	**<0.001**
SO production (nmol O_2_ per 250 000 cells)	1.91 ± 2.46	2.86 ± 3.59	0.140
PON-1 activity (µMol P-Nitrophenol/L/serum/min)	671 (418–923)	472 (213–799)	**0.045**
Endothelial function			
FMD (%)	6.9 ± 3.1	6.9 ± 3.3	0.996
Inflammatory measures			
EGF (pg/mL)	15.7 (9.2–35.3)	24.8 (14.1–52.6)	**0.031**
GRO (pg/mL)	490 (286–594)	535 (378–765)	**0.037**
PDGF-AA (ng/mL)	1035 (752–1874)	1817 (1078–2004)	**0.013**
PDGF-BB (ng/mL)	8708 (5633–10549)	9200 (7693–10493)	0.259
sCD40L (ng/mL)	3233 (1441–6411)	6228 (2478–15067)	**0.012**
Inflammatory risk score	9.2 ± 2.4	10.4 ± 2.6	**0.030**

Data are expressed as mean ± standard deviation for normally distributed data and median (interquartile range) for non-normally distributed data, respectively. Bold denotes significant difference (P<0.05).

BMI, body mass index; DBP, diastolic blood pressure; EGF, epidermal growth factor; eGFR, estimated glomerular filtration rate; FMD, flow-mediated dilation; GRO, chemokine growth-regulated oncogene; HbA_1c_, glycated haemoglobin; HDL, high-density lipoprotein; LDL, low-density lipoprotein; MAP, mean arterial pressure; PDGF-AA, platelet-derived growth factor AA; PDGF-BB, platelet-derived growth factor BB; SBP, systolic blood pressure; sCD40L, soluble CD-40 ligand.

**Table 2:** HDL levels, inflammation, and HDL functionality in adolescents with Type 1 diabetes with and without evidence of early renal dysfunction

	Low ACR, mean ± SD	High ACR, mean ± SD	Mean difference (95% CI)	*P*-value
*n*	36	34		
HDL-c (mmol/L)	1.69 ± 0.41	1.66 ± 0.35	−0.03 (−0.22 to 0.15)	0.793
HbA_1c_ (%)	8.2 ± 1.1	8.5 ± 1.1	0.3 (−0.2 to 0.9)	0.200
eGFR (mL/min/1.73 m^2^)	119 ± 17	131 ± 23	11 (2 to 21)	**0.022**
ACR (mg/mmol)	0.67 ± 0.11	2.24 ± 1.22	1.57 (1.16 to 1.97)	**<0.001**
Inflammatory risk score	9.5 ± 2.4	11.3 ± 2.5	1.8 (0.6 to 2.9)	**0.004**
FMD (%)	7.0 ± 3.4	6.8 ± 3.1	−0.2 (−1.8 to 1.4)	0.805
NO bioavailability (% change from buffer-treated cells)	33.3 ± 7.3	25.0 ± 7.7	−8 (−12 to −4)	**<0.001**
SO production (nmol O_2_ per 250 000 cells)	2.11 ± 3.49	3.95 ± 3.57	1.8 (0.1 to 3.5)	**0.048**
PON-1 activity (µMol P-Nitrophenol/L/serum/min)	564 ± 373	533 ± 407	−31 (−212 to 149)	0.735

Data are expressed as mean ± SD or mean difference (95% CI). Bold denotes significant difference (P<0.05).

ACR, albumin:creatinine ratio; CI, confidence interval; eGFR, estimated glomerular filtration rate; HbA1c, glycated haemoglobin; HDL-c, high-density lipoprotein cholesterol; NO, nitric oxide; PON-1, paraoxonase-1; SD, standard deviation; SO, superoxide.

### HDL functionality

HDL-mediated endothelial NO bioavailability (29.5 ± 8.5 vs. 38.0 ± 8.9%; *P* < 0.001) and serum PON-1 activity [472 (213–799) vs. 671 (418–923) µmol p-nitrophenol/L/serum/min; *P* = 0.045] were reduced in adolescents with Type 1 diabetes compared with controls, whereas SO production (2.86 ± 3.59 vs. 1.91 ± 2.46 nmol O_2_^−^/250 000 cells; *P* = 0.140) was similar between the two groups (*Table 1*). When further stratifying patients with Type 1 diabetes by ACR groups, NO bioavailability was found to be reduced (25 ± 8 for high ACR group vs. 33 ± 7 and 38 ± 9% for low ACR group and healthy controls, respectively; *P* < 0.001 for high ACR vs. other groups) and SO production increased (3.71 ± 3.57 vs. 2.11 ± 3.49 and 1.91 ± 2.47 nmol O_2_ per 250 000 cells; *P* < 0.05 for high-ACR vs. other groups) only in patients in the high-ACR group, with these changes independently associated with high-ACR and high-inflammation, respectively (*P* < 0.001 and *P* = 0.018; *Table 3*). No relationship was observed between any measure of HDL function and HbA1c, or with any other common risk factors such as age, sex, BMI *z*-score, or systolic blood pressure (*P* > 0.05 for all).

**Table 3:** Multivariable regression analysis showing independent predictors of HDL functionality in adolescents with Type 1 diabetes

	NO bioavailability (% change from buffer-treated cells)		SO production (nmol O_2_ per 250 000 cells)		PON-1 activity (µMol P-nitrophenol/L/serum/min)	
	Beta (95% CI)	*P*-value	Beta (95% CI)	*P*-value	Beta (95% CI)	*P*-value
ACR group (H/L)	−**9.3 (**−**13.7 to** −**4.8)**	**<0.001**	1.59 (−0.38 to 3.56)	0.112	−15 (−234 to 204)	0.892
Inflammation (H/L)	−1.4 (−5.7 to 3.0)	0.527	**2.35 (0.42 to 4.27)**	**0.018**	70 (−148 to 287)	0.523
eGFR (mL/min/1.73 m^2^)	0.1 (0.0 to 0.2)	0.093	−0.02 (−0.07 to 0.03)	0.446	0 (−6 to 6)	0.955
HbA1c (%)	0.3 (−1.7 to 2.3)	0.763	−0.19 (−1.06 to 0.69)	0.672	2 (−93 to 97)	0.966
Age (years)	−0.1 (−1.2 to 1.4)	0.887	−0.71 (−0.33 to 0.41)	0.604	−39 (−104 to 26)	0.231
Disease duration (years)	−0.2 (−0.8 to 0.4)	0.520	0.18 (−0.07 to 0.43)	0.151	13 (−16 to 42)	0.372
Sex (M/F)	0.4 (−.4.7 to 4.0)	0.861	−0.35 (−2.28 to 1.58)	0.717	79 (−141 to 300)	0.475
BMI (z-score)	−0.4 (−2.8 to 2.1)	0.765	−0.85 (−1.93 to 0.23)	0.119	23 (−102 to 148)	0.712

Bold denotes significant difference (P<0.05). ACR, albumin:creatinine ratio; BMI, body mass index; eGFR, estimated glomerular filtration rate; HbA_1c_, glycated haemoglobin; Inflammation, inflammatory risk score.

### Relationship between HDL-c levels, inflammatory burden, and HDL and endothelial function

**Figure 1:**
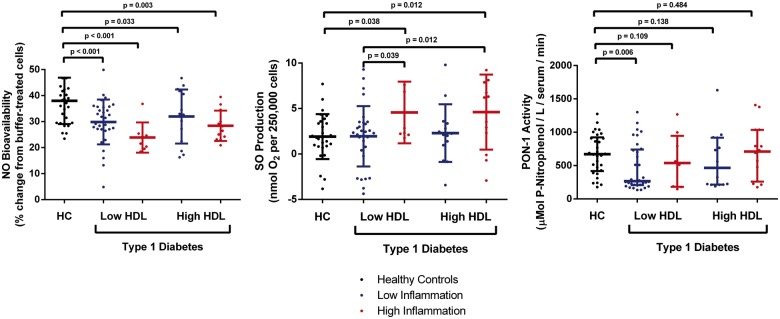
Combined effect of HDL-c levels and inflammatory burden on HDL function in adolescents with Type 1 diabetes. Data are expressed as mean ± standard deviation for NO and SO, and median (interquartile range) for PON-1. HC, healthy controls; HDL, high-density lipoprotein; NO, nitric oxide; PON-1, paraoxonase-1; SO, superoxide.

**Figure 2:**
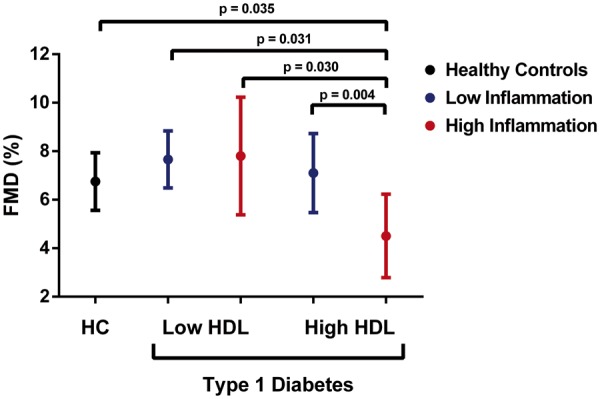
Combined effect of HDL-c levels and inflammatory burden on endothelial function in adolescents with Type 1 diabetes. Linear regression model adjusted for age, sex, body mass index *z*-score, estimated glomerular filtration rate, and systolic blood pressure. Data are expressed as mean ± 95% confidence interval. FMD, flow-mediated dilation; HC, healthy controls; HDL, high-density lipoprotein.

Due to the known interaction between HDL-c levels, inflammatory burden, and CVD outcomes in adult populations,^19^^,^^29^ we next stratified patients with Type 1 diabetes by levels of HDL concentration and inflammatory risk score (i.e. low HDL-c/low inflammation, low HDL-c/high inflammation, high HDL-c/low inflammation, high HDL-c/high inflammation), and investigated their relationship to HDL and endothelial function. NO bioavailability was found to be reduced in all groups compared with controls (*P* < 0.05 for all), whereas SO production was increased only in those with a high inflammatory score (*P* < 0.05; *Figure 1*), and PON-1 activity was reduced in patients with Type 1 diabetes and low levels of both HDL and inflammation. While FMD was similar overall between patients and controls (*Table 1*), and between ACR groups (*Table 2*), patients with a combination of high HDL-c levels and a high inflammatory score were found to have decreased FMD (4.52 ± 0.9%) compared with all other groups (6.97 ± 0.63, 7.7 ± 0.6, 7.1 ± 0.8, and 7.7 ± 1.2%; *P* < 0.05 for all; *Figure 2*). Further analyses identified these patients as being predominantly female (86%; *P* = 0.021; Supplementary material online, *Table S2*) and belonging to the high-risk ACR group (71%; *P* = 0.020; Supplementary material online, *Table S3*).

**Take home figure:**
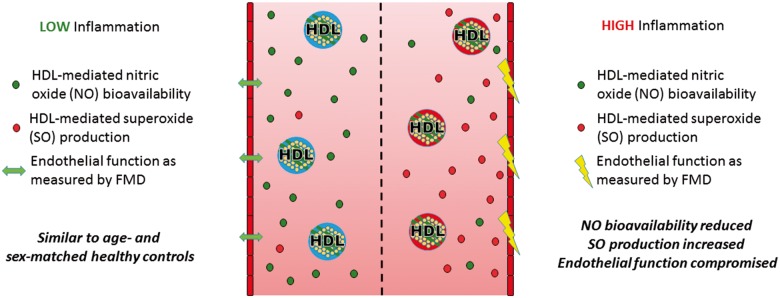
Elevated levels of HDL-c are common in young people with type 1 diabetes, but may be detrimental to vascular health in the presence of increased systemic inflammation.

## Discussion

This study is the first to investigate HDL functionality and its relationship to vascular health in adolescents with Type 1 diabetes. We have demonstrated that systemic inflammation accompanying early signs of renal dysfunction—but not glycaemic control—is associated with a dysfunctional HDL phenotype even at this early age. These changes in the HDL phenotype were characterized by a decreased capacity for HDL to stimulate endothelial NO bioavailability and an increased capacity for SO anion generation. A combination of elevated HDL-c and systemic inflammation was associated with impairment in endothelial function, with this phenotype found predominantly in patients with Type 1 diabetes and early elevations in ACR. These individuals may therefore represent a high-risk group for accelerated development of CVD, even from the second decade of life.

Current guidelines recommend measuring HDL-c for risk estimation and before initiation of lipid-lowering therapy.^30^ Patients with Type 1 diabetes commonly maintain or even increase HDL-c levels in comparison to the general population^9–11^^,^^13^; findings which were confirmed in the current study. Whether these increases are associated with cardio-protection in this clinical population, however, remains contentious. While a number of studies have shown inverse associations between HDL levels and the development of renal and vascular damage in adults with the disease,^31–33^ others have shown that very high levels of HDL may be associated with adverse—rather than positive—outcomes.^34^ In addition, both animal and human studies have shown the presence of numerous detrimental changes in HDL composition and function in the presence of Type 1 diabetes,^8^ with these changes—rather than serum concentration of HDL—predictive of future cardiovascular risk.^35^ Although studies in young people with the disease are limited, a number of recent studies have indicated that evidence of HDL dysfunction may be evident even at this early age, with changes such as reduced PON-1 activity,^36^ increased lipoprotein-associated phospholipase A2,^37^ and reduced reverse cholesterol transport^38^ reported.

In the current study, we chose to examine changes in a number of endothelial-protective functions of HDL—namely HDL-mediated endothelial NO bioavailability, SO production, and activity of the anti-oxidant enzyme PON-1. The reasons for selecting these functional changes are three-fold. Firstly, these changes have previously been shown to be related to endothelial dysfunction in patients with Type 2 diabetes.^16^ Secondly, endothelial dysfunction is one of the earliest signs of subclinical vascular damage detectable in the young and has repeatedly been shown to be present in children and adolescents with Type 1 diabetes.^4^^,^^6^^,^^7^ Lastly, previous work from our group has shown that systemic inflammation and renal dysfunction—both of which we have also previously shown to be present in adolescent cohorts^20^^,^^23^—can result in adverse changes in these HDL phenotypes in other adult and adolescent populations.^17^^,^^18^

Inflammation is a heterogenous condition involving a myriad of inflammatory cytokines and chemokines but is typically monitored clinically through the use of single inflammatory markers such as interleukin-6 (IL-6) and Tumour necrosis factor alpha (TNFα), or downstream acute phase proteins such as high-sensitivity C-reactive protein. These single measures may not accurately represent overall inflammatory risk, especially in younger cohorts where risk factor profiles and accompanying comorbidities are different from older individuals. In order to more accurately characterize inflammatory risk in this young patient cohort, we selected five pro-inflammatory cytokines previously shown by our group to be elevated in adolescents with Type 1 diabetes,^20^ and used these to create a disease-specific inflammatory risk score. In support of the value of this approach, we replicated our previous findings in adolescents with Type 1 diabetes, observing elevated levels of all five inflammatory markers chosen for our risk score, but no observable differences in IL-6 or TNF-α levels (*P* = 0.851 and 0.224; data not shown). Microalbuminuria (MA) is the most commonly-used risk marker for early renal dysfunction in adults with Type 1 diabetes, but its use in adolescents is challenging due to the frequent appearance of transient MA during puberty that subsequently resolves upon progression to adulthood.^39^ In order to assess the earliest signs of renal dysfunction at this young age, we used a standardized measure of ACR adjusted for age, sex, and diabetes duration, previously developed by our group^22^ and shown to predict risk of multiple cardio-renal complications even when ACR levels are within the normal range.^4^^,^^22^^,^^23^^,^^27^ Using these measures, we observed evidence of HDL dysfunction only in patients with Type 1 diabetes and elevated levels of both inflammation and ACR. Multivariable regression analysis identified ACR as being independently related to HDL-mediated NO bioavailability, while SO production was found to relate to increased inflammatory risk score. Interestingly, no association was observed between any measure of HDL function and HbA1c; supporting previous research in adults in which a lowering of Hba1c levels were shown to have no impact on HDL function.^40^

Given the well-established reverse association between inflammation, dysfunctional HDL, and CVD morbidity and mortality,^19^^,^^29^ we next examined whether the presence of a high inflammatory risk score in adolescent patients with Type 1 diabetes altered the relationship between circulating HDL levels and early subclinical markers of vascular health. Due to a lack of hard clinical endpoints in the young, we chose FMD as our surrogate marker of subclinical disease due to its widespread prevalence in this patient population,^4^^,^^6^^,^^7^ as well as its established ability to predict future adverse CVD events.^41^ While previous studies in this age group have reported a modest decrease in FMD (∼1%) in adolescents with Type 1 diabetes compared with healthy controls,^4^ we observed no overall difference in this particular cohort. However, when stratifying patient by levels of HDL and inflammation, we observed a substantially greater decrease in FMD (∼2.5–3%) occurring only in patients with a combination of high HDL levels (range 1.5–2.5 mmol/L) and a high inflammatory risk score, when compared with all other groups. These changes occurred predominantly in the group with elevated ACR and were accompanied by evidence of decreased HDL-mediated NO bioavailability and increased SO production. These findings suggest that in this high-risk group of adolescents, increased levels of HDL and systemic inflammation may interact to compromise vascular health.

This study is not without its limitations. Firstly, results from this study suggested that adolescent females with Type 1 diabetes were those most likely to have a high HDL/high inflammation phenotype, and therefore, decreased FMD. These findings are similar to those reported previously in which adult females with Type 1 diabetes and high levels of HDL-c were found to be at an elevated risk for coronary artery disease.^34^ This study was not powered to investigate sex-specific outcomes, however, and further research in this field is therefore warranted. Due to the cross-sectional nature of the study, it is not possible to determine the direction of causality between the observed associations, and further interventional studies will be required in order to address these issues. Such studies might include the study of the incorporation of acute phase proteins such as serum amyloid-a,^19^ and symmetric dimethylarginine^29^ into the HDL particle, as these are widely believed to underlie compositional and functional changes and are commonly used to stratify inflammatory risk. Although these additional specific measures were not included in the study design, we still believe our results are robust and the findings should inform future evaluation of HDL levels in young people with Type 1 diabetes. The addition of a pre-established age- and disease-specific inflammatory risk score could also provide a sensitive measure of systemic inflammation in this cohort. Likewise, the use of a standardized ACR measure designed and validated by our group^22^ would allow further investigation of associations between early changes in renal function and HDL function that would not be possible using adult-specific measures such as MA.

In conclusion, this study shows for the first time that early changes in renal function (assessed by ACR) and chronic inflammation are associated with decreased HDL functionality in adolescents with Type 1 diabetes, and that these changes are related to early signs of vascular damage when HDL-c levels are also high. These findings suggest that increased HDL levels, a common finding in Type 1 diabetes, may be detrimental to vascular health when accompanied by renal dysfunction and chronic inflammation.

## Supplementary Material

ehz114_Supplementary_Data

## Notes

**See page 3567 for the editorial comment on this article (doi: 10.1093/eurheartj/ehz502)**
