# Supplementary material for: Elevated high-density lipoprotein in adolescents with Type 1 diabetes is associated with endothelial dysfunction in the presence of systemic inflammation
Source: Eur Heart J. 2019 Mar 12;40(43):3559–66. doi: 10.1093/eurheartj/ehz114 (PMC6855140; doi:10.1093/eurheartj/ehz114)
Supplement: ehz114_Supplementary_Data [file ehz114_supplementary_data.docx]

**SUPPLEMENTARY FILE**

**Chiesa et al.**

**Elevated high-density lipoprotein in adolescents with type 1 diabetes is associated with endothelial dysfunction in the presence of systemic inflammation**

**SUPPLEMENTARY METHODS**

***Participant Recruitment***

Patients in this study consisted of a subsample of individuals from Toronto, Canada, who were originally screened and approached to take part in the Adolescent type 1 Diabetes cardio-renal Intervention Trial (AdDIT) – full details of which have been previously published (1–4). Inclusion criteria for all patients were 1) age 10–16 years, 2) type 1 diabetes diagnosis for more than 1 year or C-peptide negative, and 3) centralised assessment of ACR based on six early morning urines and adjusted for age, sex, age at diagnosis, and duration of disease. Exclusion criteria were 1) other types of diabetes, 2) severe hyperlipidaemia and family history data to support diagnosis of familial hypercholesterolaemia, 3) established hypertension unrelated to diabetic nephropathy, 4) prior exposure to the investigational products (statins and ACE inhibitors), 5) other comorbidities considered unsuitable by the investigator (excluding treated hypothyroidism and coeliac disease), and 6) proliferative retinopathy. Based on previous work from our group showing an increased risk of early adverse CV phenotypes in patients with an elevated ACR, we opted to recruit equal numbers of participants from high- and low-risk ACR groups, described previously (5), alongside healthy age- and sex-matched controls. Power calculations suggested the need for 40 patients in each group in order to have 80% power to detect a 2 nmol O_2_^-^/250,000 cells difference in HDL-mediated SO production – the most variable of the HDL functionality measures – using a two-sided 5% level of significance. Accordingly, forty high-risk patients were selected from an ancillary cohort of participants who refused to participation in the AdDIT RCT, but instead consented to being followed on an observational basis. Any patients with missing data (HDL functionality, inflammatory assays, or FMD) were excluded, and the remainder were age- and sex-matched to low-risk and healthy control participants in order to conduct the current study.

***HDL Function***

*Nitric oxide bioavailability*: The effect of HDL on endothelial NO bioavailability (HDL 50 µg/ml; 60 min, 37 °C) was measured using endothelial cells (bovine aortic endothelial cells BAEC; passage 4-7; Lonza Bio Science) incubated with 4,5-diaminofluorescein diacetate (DAF-2; 1 µM; Cayman Chemical), and triazolo- fluorescein fluorescence. NO bioavailability was quantified using a fluorescence protocol with an excitation wavelength of 485 nm using a FLUOstar Omega microplate reader (BMG Labtech) (6). Each assay was performed in duplicate and data are expressed as percent change versus buffer-treated cells.

*Superoxide production*: The effect of HDL on endothelial cell superoxide (SO) production was measured in TNFα-stimulated (5 ng/ml, R&D Systems) human aortic endothelial cells (HAEC) by ESR spectroscopy. Briefly, HAEC were incubated with HDL (50 µg/ml, 60 min, 37 °C) and TNFα, and re-suspended in Krebs-Hepes buffer (pH 7,4; Noxygen) containing diethyldithiocarbamic acid sodium salt (5 µM, Noxygen) and deferoxamine methanesulfonate salt (25 µM, Noxygen). ESR spectra were recorded after addition of the spin probe 1-hydroxy- 3-methoxycarbonyl-2,2,5,5-tetramethylpyrrolidine (CMH; Noxygen; final concentration 200 µM) using a Bruker e-scan spectrometer (Bruker Biospin). The ESR instrumental settings were as follows: centre field (B0) 3495 G; field sweep width 10 G; microwave frequency 9.75 GHz; microwave power 19.91 mW; magnetic field modulation frequency 86.00 kHz; modulation amplitude 2.60 G; conversion time 10.24 msec; number of x-scans 1020 (7). Each assay was performed in duplicate and data are expressed as the absolute amount of SO produced relative to buffer treated cells (nmol O_2_^-^/250,000 cells).

### Paraoxonase Activity: Serum paraoxonase (PON-1) activities were measured by UV spectrophotometry in a 96-well plate format using paraoxon (SigmaeAldrich, St Louis, Missouri). Briefly, 10 µL of serum was diluted in a reaction mixture containing 10 mM Tris hydrochloride (pH 8.0), 1 M sodium chloride and 2 Mm calcium chloride. At 24 °C, 1.5 mM paraoxon was added to initiate the reaction, and the increase in absorbance at 405 nm was recorded over 30 min using a FLUOstar Omega microplate reader (BMG Labtech). An extinction coefficient of 17.000 M^-1^ cm^-1^ (at 24 °C) was used to calculate units of PON-1 activity (8). Each assay was performed in triplicate and data are expressed as µmol p-nitrophenol/L/serum/min.

***Endothelial Function***

Each participant rested in a supine position for 10 min before an optimised longitudinal ECG-gated image of the right brachial artery was obtained by high-resolution ultrasound (Vivid7, GE Healthcare) using a 12MHz probe. The test began with 5 min of rest, followed by 5 min of ischaemia stimulated by inflation of a pneumatic cuff around the forearm at 300 mmHg. Reactive hyperaemia was induced after cuff release and measurements continued for 5 min. The change in diameter of the brachial artery was measured offline using an automatic edge-detection algorithm (Brachial Tools, Medical Imaging Applications, Coralville, IA). FMD was determined as the relative change from baseline diameter (%) following cuff release. Intra-session intra-observer variability from the Toronto site showed a mean difference in FMD of 0.1% with a COV of 16%, whereas inter-observer variability showed a mean difference in FMD of 0.9% with a COV of 26%. Based on power curves previously generated by our group (9), we calculated that the current study would provide 80% power to detect a difference in FMD of ~ 1-2% between groups with a two-sided 5% level of significance (based on a parallel study design including 15-30 participants per group and assuming a measurement COV of between 15-25%).

**SUPPLEMENTARY TABLES**

|  | **Healthy Controls** | **Type 1 Diabetes** | **p value** |
| --- | --- | --- | --- |
|  |  |  |  |
| EGF | 15.7 (9.2 – 35.3) | 24.8 (14.1 – 52.6) | **0.031** |
| GRO | 490 (286 – 594) | 535 (378 – 765) | **0.037** |
| sCD40L | 3233 (1441 – 6411) | 6228 (2478 – 15067) | **0.012** |
| PDGF-AA | 1035 (752 – 1874) | 1817 (1078 – 2004) | **0.013** |
| PDGF-BB | 8708 (5633 – 10549) | 9200 (7693 – 10493) | 0.259 |
| FGF2 | 36.5 (24.5 – 68.1) | 37.4 (23.9 – 64.5) | 0.807 |
| Eotaxin | 66.8 (49.4 – 84.3) | 81.7 (54.3 – 105.5) | 0.106 |
| TGF-a | 5.3 (1.8 – 8.2) | 4.4 (2.5 – 7.7) | 0.886 |
| G-CSF | 27.4 (21.3 – 35.1) | 29.8 (17.7 – 41.4) | 0.665 |
| Flt-3L | 11.1 (4.3 – 66.5) | 5.0 (1.1 – 21.2) | 0.085 |
| GM-CSF | 19.0 (13.7 – 25.9) | 18.6 (12.0 – 29.6) | 0.789 |
| Fractalkine | 51.3 (2.9 – 76.0) | 15.8 (3.0 – 75.2) | 0.494 |
| IFNa2C | 34.0 (16.0 – 84.3) | 26.5 (13.2 – 54.7) | 0.275 |
| IFNy | 17.4 (5.5 – 46.7) | 10.4 (4.8 – 32.7) | 0.392 |
| IL-10 | 5.2 (3.1 – 11.7) | 6.0 (3.2 – 12.8) | 0.855 |
| MCP-3 | 29.1 (20.4 – 87.7) | 35.9 (16.7 – 69.1) | 0.504 |
| IL-12P40 | 43.3 (7.2 – 118.1) | 56.5 (18.8 – 141.6) | 0.260 |
| MDC | 1222.7 (924.5 – 1587.2) | 1201.4 (949.0 – 1552.3) | 0.813 |
| IL12-P70 | 4.4 (2.9 – 7.6) | 4.3 (2.5 – 9.8) | 0.876 |
| IL-13 | 8.3 (4.0 – 24.5) | 9.7 (3.5 – 16.8) | 0.870 |
| IL-15 | 6.0 (0.8 – 14.9) | 5.6 (1.2 – 15.9) | 0.787 |
| IL-17A | 10.7 (2.7 – 25.6) | 4.3 (2.4 – 14.5) | 0.174 |
| IL-1RA | 30.1 (15.9 – 68.1) | 24.9 (13.5 – 49.5) | 0.332 |
| IL-1a | 28.8 (6.2 – 92.7) | 21.4 (5.8 – 53.3) | 0.396 |
| IL-9 | 1.9 (1.2 – 4.3) | 2.1 (1.2 – 5.1) | 0.498 |
| IL-1B | 5.1 (1.1 – 10.1) | 6.2 (1.9 – 14.7) | 0.453 |
| IL-2 | 3.4 (0.7 – 9.7) | 4.2 (1.5 – 11.2) | 0.438 |
| IL-3 | 0.9 (0.2 – 1.7) | 0.7 (0.2 – 1.8) | 0.696 |
| IL-4 | 11.9 (1.9 – 38.5) | 13.2 (2.2 – 43.0) | 0.626 |
| IL-5 | 0.6 (0.3 – 1.2) | 0.6 (0.2 – 1.3) | 0.589 |
| IL-6 | 3.0 (1.0 – 6.8) | 3.2 (1.5 – 6.6) | 0.570 |
| IL-7 | 2.9 (2.4 – 4.1) | 3.7 (2.6 – 4.3) | 0.204 |
| IL-8 | 25.0 (12.1 – 37.2) | 18.2 (11.0 – 26.8) | 0.220 |
| **IP-10** | **119.0 (99.7 – 161.3)** | **96.1 (72.0 – 138.4)** | **0.015** |
| MCP-1 | 326.4 (221.7 – 424.7) | 329.1 (244.1 – 449.7) | 0.489 |
| MIP-1a | 9.1 (7.0 – 15.6) | 12.5 (6.8 – 18.0) | 0.195 |
| MIP-1b | 48.5 (32.7 – 72.3) | 40.9 (30.2 – 65.6) | 0.359 |
| RANTES | 1669.2 (1195.6 – 2602.1) | 1737.8 (1014.5 – 2818.2) | 0.622 |
| TNF-a | 8.0 (6.2 – 9.6) | 9.4 (6.0 – 13.2) | 0.128 |
| TNF-B | 24.5 (7.5 – 105.5) | 26.9 (10.0 – 62.1) | 0.933 |
| VEGF | 81.7 (34.4 – 195.2) | 84.8 (44.6 – 161.1) | 0.679 |

**Supplementary Table 1: Additional cytokine/chemokine levels in healthy controls and patients with type 1 diabetes as measured by the Discovery Human Cytokine Array / Chemokine Array 41-Plex Assay**

Data represent median (IQR). Differences between groups assessed using Kruskall-Wallis test. Significant difference classed as p < 0.05. Abbreviations: EGF, epidermal growth factor; GRO, growth-regulated oncogene; sDC40L, soluble CD40 ligand; PDGF, platelet-derived growth factor; FGF, fibroblast growth factor; TGF, transforming growth factor; G-CSF, granulocyte colony-stimulating factor; Flt-3L, FMS-like tyrosine kinase 3 ligand; GM-CSF, granulocyte macrophage colony-stimulating factor, IFNa2c, interferon alpha 2; IFNy, interferon gamma; IL, interleukin; MCP, monocyte chemotactic protein; MDC, macrophage-derived chemokine; IP-10, interferon gamma-induced protein 10; MIP, macrophage inflammatory protein; RANTES, regulated on activation, normal T-cell secreted and expressed; TNF, tumour necrosis factor; VEGF, vascular endothelial growth factor.

**Supplementary Table 2: Sex distribution within combined HDL/inflammation groups**

|  | **Female**  **n (%)** | **Male**  **n (%)** |
| --- | --- | --- |
|  |  |  |
| Healthy Controls | 14 (47) | 16 (53) |
| Low HDL / Low Inflammation | 16 (48) | 17 (42) |
| Low HDL / High Inflammation | 5 (63) | 3 (37) |
| High HDL / Low Inflammation | 10 (67) | 5 (33) |
| High HDL / High Inflammation | 12 (86) | 2 (14) |

Abbreviation – HDL, high-density lipoprotein

|  | **High-Risk ACR**  **n (%)** | **Low-Risk ACR**  **n (%)** |
| --- | --- | --- |
|  |  |  |
| Low HDL / Low Inflammation | 13 (39) | 20 (61) |
| Low HDL / High Inflammation | 6 (75) | 2 (25) |
| High HDL / Low Inflammation | 5 (33) | 10 (67) |
| High HDL / High Inflammation | 10 (71) | 4 (29) |

**Supplementary Table 3: Proportion of high- and low-risk ACR patients per HDL/inflammation group**

Abbreviation – HDL, high-density lipoprotein

**Supplemental References**

1. Bryden K, Dunger D, Mayou R, et al. Adolescent type 1 Diabetes cardio-renal Intervention Trial (AdDIT). BMC Pediatr. 2009;9:79.

2. Marcovecchio ML, Woodside J, Jones T, et al. Adolescent Type 1 Diabetes Cardio-Renal Intervention Trial (AdDIT): Urinary screening and baseline biochemical and cardiovascular assessments. Diabetes Care 2014;37:805–813.

3. Marcovecchio ML, Chiesa ST, Bond S, et al. ACE Inhibitors and Statins in Adolescents with Type 1 Diabetes. N. Engl. J. Med. 2017;377:1733–1745.

4. Marcovecchio ML, Chiesa ST, Armitage J, et al. Renal and Cardiovascular Risk According to Tertiles of Urinary Albumin-to-Creatinine Ratio: The Adolescent Type 1 Diabetes Cardio-Renal Intervention Trial (AdDIT). Diabetes Care 2018:dc181125.

5. Dunger DB, Schwarze CP, Cooper JD, et al. Can we identify adolescents at high risk for nephropathy before the development of microalbuminuria? Diabet. Med. 2007;24:131–6.

6. Besler C, Heinrich K, Rohrer L, et al. Mechanisms underlying adverse effects of HDL on eNOS-activating pathways in patients with coronary artery disease. J. Clin. Invest. 2011;121:2693–708.

7. Sorrentino SA, Besler C, Rohrer L, et al. Endothelial-vasoprotective effects of high-density lipoprotein are impaired in patients with type 2 diabetes mellitus but are improved after extended-release niacin therapy. Circulation 2010;121:110–22.

8. Bhattacharyya T, Nicholls SJ, Topol EJ, et al. Relationship of paraoxonase 1 (PON1) gene polymorphisms and functional activity with systemic oxidative stress and cardiovascular risk. JAMA 2008;299:1265–76.

9. Charakida M, de Groot E, Loukogeorgakis SP, et al. Variability and reproducibility of flow-mediated dilatation in a multicentre clinical trial. Eur. Heart J. 2013;34:3501–3507.
